# Three Decades of Spinal Cord Injury in Saudi Arabia: Trends in Incidence, Prevalence, and Disability Outcomes

**DOI:** 10.3390/jcm14248836

**Published:** 2025-12-13

**Authors:** Ahmad F. Alahmary, Mishal M. Aldaihan, Vishal Vennu, Saad M. Bindawas

**Affiliations:** 1 Department of Physical Therapy, College of Applied Medical Sciences, Qassim University, Buraydah 52571, Saudi Arabia; a.alahmary@qu.edu.sa; 2 Department of Rehabilitation Sciences, College of Applied Medical Sciences, King Saud University, Riyadh 11451, Saudi Arabia; mishaldaihan@ksu.edu.sa (M.M.A.); vvennu@ksu.edu.sa (V.V.)

**Keywords:** disability, transport injury, fall, unintentional injury

## Abstract

**Background/Objective:** Spinal cord injury (SCI) is a life-altering condition representing a major cause of long-term disability and substantial health burden worldwide. In the Middle East, including Saudi Arabia, rapid urbanization and evolving injury patterns may have influenced SCI trends; however, national data remain limited. This study aimed to examine age-standardized trends in SCI incidence, prevalence, and years lived with disability (YLDs) in Saudi Arabia from 1990 to 2021, comparing transport-related and non-transport unintentional injuries, and describing age- and sex-specific SCI patterns in 2021. **Methods:** Using data from the Global Burden of Diseases (GBD) 2021 study, we conducted a population-based trend analysis for Saudi Arabia from 1990 to 2021, stratified by age, sex, and injury cause. Outcomes included age-standardized incidence, prevalence, and YLD rates per 100,000 population, along with percentage changes, average annual percentage changes, and rate ratios with 95% uncertainty intervals (UIs). **Results:** Between 1990 and 2021, age-standardized SCI showed a point estimate increase in incidence (25.0%; 95% UI: −28.3 to 116.8) and prevalence (24.3%; 95% UI: 0.8 to 53.4), while YLDs showed a modest rise (1.4%; 95% UI: −44.5 to 83.9). Males experienced greater increases in incidence (31.9%) and prevalence (32.3%) than females. Non-transport unintentional injuries surpassed transport-related causes, accounting for nearly 75% of SCI-related YLDs in 2021. The highest burden occurred among young adult males (highest incidence) and older adults (peak prevalence). **Conclusions:** The burden of SCI in Saudi Arabia has increased over the past three decades, with a shift toward non-transport unintentional injuries. Because wide uncertainty intervals limit definitive conclusions on trend direction, strengthening injury prevention, rehabilitation, and surveillance programs is crucial to mitigate this growing burden.

## 1. Introduction

Spinal cord injury (SCI) is a life-altering neurological condition and a major cause of long-term disability and premature mortality worldwide [1,2]. In 2019, approximately 20.6 million individuals were living with SCI globally, contributing to 6.2 million years lived with disability (YLDs), with minimal change in global age-standardized incidence or prevalence since 1990 [3]. Trauma remains the dominant etiology of SCI worldwide [4]. Road traffic crashes and falls account for 40–50% and 20–30% of cases, respectively, while non-traumatic causes, including degenerative and infectious diseases, comprise 10–25%, with substantial regional variation [5]. SCI imposes a significant economic burden in high-income countries [6], with annual direct medical costs exceeding $3 billion in the United States alone. Indirect productivity losses exacerbate this financial strain, particularly in low- and middle-income countries [3,7].

Saudi Arabia faces substantial public health challenges related to traffic-related injuries [8], compounded by SCI [9]. The country recorded 28.8 road traffic deaths per 100,000 population in 2016, nearly triple the global average of 10 per 100,000 [10]. Multiple hospital-based studies from Riyadh and other Saudi cities document that motor vehicle collisions account for approximately 80–85% of traumatic SCI cases, predominantly among young males, representing the leading cause of complete spinal paralysis. Reported rates include 80.1% at Prince Sultan Military Medical City, 82.6% at Prince Mohammed bin Abdul Aziz Hospital, and 85% at Riyadh Military Hospital [11,12,13]. However, these hospital-based estimates may reflect selection bias toward acute trauma centers and may span 2003 to 2018. Vision 2030 traffic safety reforms, including automated speed camera enforcement and enhanced trauma care systems, have been implemented since 2016 [14]. Preliminary World Health Organization (WHO) data suggest that road traffic fatalities declined by 35% between 2016 and 2021; however, the specific impact on SCI incidence remains to be systematically evaluated. The historical prohibition on women driving, which resulted in fewer female SCI cases from road traffic crashes [15], was lifted in 2017, although its impact on female injury patterns remains undocumented.

Non-transport unintentional injuries, particularly falls, represent a growing concern. Falls from construction heights among occupational workers and low-energy falls in older adults constitute an increasing proportion of SCI cases [16]. Saudi Arabia’s population structure is undergoing a rapid transition, with life expectancy increasing from 70.6 years in 2000 to 76.4 years in 2021, thereby expanding the population at risk of age-related falls [10]. This demographic transition, combined with substantial construction and infrastructure expansion under Vision 2030 economic diversification initiatives, creates converging risk factors for non-transport SCI.

Despite several hospital-based studies documenting SCI patterns in Saudi Arabian trauma centers [11,12,13,15] and a recent systematic review synthesizing evidence from 2356 traumatic SCI cases across nine studies [17], significant knowledge gaps persist. Hospital-based data are constrained by selection bias toward acute trauma centers, temporal limitations (most studies span 2003–2018), and the absence of population-level denominators, which prevents accurate incidence calculation. The 2024 systematic review by Almallah and colleagues confirmed the predominance of motor vehicle accidents and male predominance in facility-based samples. Still, it could not assess population-level trends or age-standardized burden due to heterogeneous study designs [17]. Furthermore, no population-based analysis has examined three-decade national trends using the standardized Global Burden of Disease (GBD) methodology, which enables international comparisons, cause-specific stratification, and alignment with global health metrics. The absence of a national SCI registry compounds these limitations, hindering comprehensive surveillance, policy evaluation, and resource allocation [18].

To address these gaps, we leveraged GBD 2021 data [19,20] to achieve four primary objectives: first, to quantify age-standardized incidence, prevalence, and YLD trends in Saudi Arabia from 1990 to 2021 with sex-specific stratification; second, to compare temporal patterns between transport-related and non-transport unintentional SCI causes; third, to characterize age- and sex-specific burden distributions in 2021; and fourth, to contextualize findings within Saudi Arabia’s Vision 2030 health transformation framework and provide evidence-based policy recommendations. The GBD 2021 framework provides a standardized estimation methodology that enables international comparisons and the assessment of Saudi Arabia’s position relative to global and regional SCI burden [19,20].

## 2. Materials and Methods

This study adheres to the Guidelines for Accurate and Transparent Health Estimates Reporting (GATHER) [21] and the Strengthening the Reporting of Observational Studies in Epidemiology (STROBE) guideline [22]. A completed GATHER checklist is provided as Supplementary Table S1.

### 2.1. Study Design and Data Source

We conducted a population-based trend analysis examining SCI estimates from the GBD 2021 study for Saudi Arabia, stratified by sex, etiology (transport versus non-transport unintentional injuries), and five-year age cohorts (10–14 to ≥55 years). Age-specific and age-standardized SCI incidence, prevalence, and YLD rates for Saudi Arabia were extracted from the publicly accessible GBD 2021 Results Tool (https://vizhub.healthdata.org/gbd-results/, accessed on 20 October 2025) [23]. The GBD 2021 study estimated the burden of 371 diseases and injuries across 204 countries and territories from 1990 to 2021 using a standardized methodology [19,23]. Data were derived from published reports, systematic reviews, vital registration systems, hospital records, survey data, and contributions from GBD collaborators, with annual updates ensuring data accuracy and reliability [5].

The GBD 2021 estimates for Saudi Arabia were derived using Disease Modeling Meta-Regression (DisMod-MR) 2.1, a Bayesian modeling framework that synthesizes multiple data sources, including published literature, hospital records, vital registration systems, and survey data. For Saudi Arabia specifically, data inputs included Saudi Ministry of Health reports, published hospital-based SCI studies, regional North African and Middle Eastern pattern data, and global covariates. Where Saudi-specific primary data were limited, estimates were informed by regional patterns adjusted for country-particular covariates, including healthcare access, injury risk factors, and demographic characteristics. The modeling approach ensures internal consistency among incidence, prevalence, remission, and excess mortality while propagating uncertainty from data sparsity through 95% uncertainty intervals (UIs).

### 2.2. Case Definitions and Classification

GBD 2021 classifies SCI as a nature of injury using the International Classification of Diseases (ICD) 9th and 10th revision editions. SCI is defined by ICD codes ICD-N-33 and ICD-N-34 as spinal cord lesions at or below the cervical level, leading to partial or total paralysis depending on injury level and severity [19]. GBD 2021 categorizes SCI into at-neck and below-neck levels. Traumatic SCI cases were subdivided by external cause into transport-related versus non-transport unintentional injuries based on ICD-10 codes. Transport-related injuries (ICD-10 V00–V99) encompass accidents involving vehicles, including motor vehicle collisions, bicycle accidents, and pedestrian injuries [2]. Non-transport unintentional injuries include primarily falls (ICD-10 W00–W19) and other accidental injuries (ICD-10 X00–X59). Intentional causes, such as violence and self-harm, as well as non-traumatic etiologies, were excluded because they represent a small fraction of cases in Saudi Arabia and fall beyond this study’s scope.

### 2.3. Outcome Measures

The primary outcomes were age-standardized incidence, prevalence, and YLD rates per 100,000 population from 1990 to 2021. Incidence is the number of new SCI cases per year. Prevalence quantifies the total number of individuals living with SCI at a given time. YLDs measure the burden of non-fatal health outcomes by multiplying prevalence by disability weights that reflect the severity of functional limitation associated with SCI. Age standardization was performed using the GBD 2021 global age structure as the reference population, allowing for comparisons across populations with different age distributions [19]. Secondary outcomes included absolute percentage changes from 1990 to 2021, average annual percentage changes, and male-to-female rate ratios. All estimates are reported with 95% UIs, which reflect uncertainty arising from data availability, modeling assumptions, and parameter estimation.

GBD 2021 categorizes causes into four hierarchical levels. Level 1 categorizes diseases into communicable, maternal, neonatal, nutritional, non-communicable, and injuries. Level 2 identifies 22 causes, with more detailed levels 3 and 4 as previously specified [19,24,25]. SCI is a level 1 cause, with specific causes reported from injury-specific level 2 categories, including transport-related and non-transport unintentional injuries.

### 2.4. Statistical Analysis

The percentage change was calculated as the relative difference between the 2021 and 1990 estimates divided by the 1990 estimate, multiplied by 100. Average annual percentage change was derived by dividing the total percentage change by the number of years in the observation period. Male-to-female rate ratios were computed by dividing male rates by female rates for each year and age group. Two rates were considered statistically different if their 95% UIs did not overlap. All analyses were conducted using the extracted GBD 2021 data without additional statistical modeling. Data visualization and descriptive analyses were performed using Microsoft Excel 2021 (Microsoft Corporation, Redmond, WA, USA).

### 2.5. Ethical Considerations

This study used de-identified, publicly available, aggregated population estimates from the GBD 2021 database, with no access to individual-level data. According to international guidance on secondary analyses of anonymized data, institutional review board approval and patient consent were not required. All primary data sources included in the GBD framework obtained the necessary ethical approvals before contributing data.

## 3. Results

### 3.1. Trend of SCI Burden in Saudi Arabia

**Figure 1:**
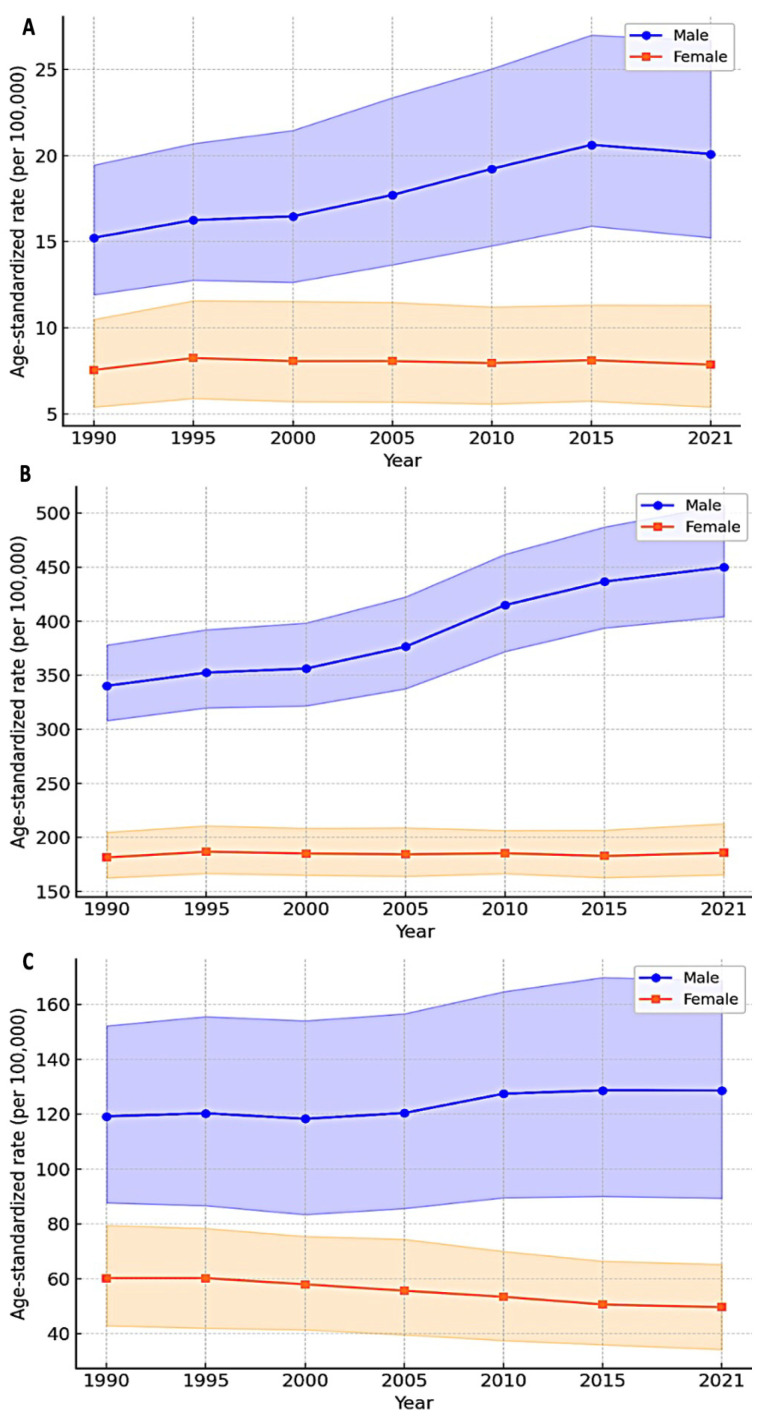
Age-standardized spinal cord injury burden in Saudi Arabia, 1990–2021. (**A**) Incidence rates per 100,000 population. (**B**) Prevalence rates per 100,000 population. (**C**) Years lived with disability (YLD) rates per 100,000 population. Data are presented for both sexes combined with 95% uncertainty intervals.

Between 1990 and 2021, the age-standardized incidence rate of SCI in Saudi Arabia increased from 12.1 per 100,000 population (95% UI: 9.4 to 15.7) to 15.1 per 100,000 (95% UI: 11.3 to 20.3), representing a 25.0% increase (95% UI: −28.3 to 116.8) over the three decades. The age-standardized prevalence rate increased from 278.2 per 100,000 (95% UI: 253.4 to 310.0) in 1990 to 345.9 per 100,000 (95% UI: 312.4 to 388.7) in 2021, representing a 24.3% increase (95% UI: 0.8 to 53.4). Age-standardized YLDs increased from 87.5 per 100,000 (95% UI: 60.5 to 123.2) in 1990 to 88.7 per 100,000 (95% UI: 61.1 to 125.9) in 2021, representing a modest 1.4% increase (95% UI: −44.5 to 83.9). Detailed temporal trends are presented in Table 1 and Figure 1.

The average annual percentage change in age-standardized incidence was 0.75% (95% UI: −1.16 to 2.50), indicating a gradual increase over the study period. Similarly, the average annual percentage change in prevalence was 0.72% (95% UI: 0.02 to 1.41), demonstrating a steady upward trajectory. In contrast, YLDs exhibited minimal average annual change at 0.04% (95% UI: −1.96 to 2.05), suggesting relative stability in disability burden despite increases in incidence and prevalence.

### 3.2. Sex-Specific SCI Trends

Males consistently demonstrated higher SCI burden across all metrics compared with females throughout the observation period. In 2021, the age-standardized incidence rate among males was 17.6 per 100,000 (95% UI: 13.0 to 23.9), more than double the rate among females at 8.6 per 100,000 (95% UI: 6.3 to 11.6), yielding a male-to-female rate ratio of 2.05 (95% UI: 1.88 to 2.23). Similarly, the prevalence rate among males in 2021 was 383.7 per 100,000 (95% UI: 285.0 to 507.5) compared with 189.7 per 100,000 (95% UI: 139.8 to 252.5) among females, with a rate ratio of 2.02 (95% UI: 1.90 to 2.15). YLDs followed a comparable pattern, with males experiencing 117.4 per 100,000 (95% UI: 80.7 to 167.7) versus 57.4 per 100,000 (95% UI: 39.5 to 81.9) among females, yielding a rate ratio of 2.04 (95% UI: 1.91 to 2.19).

Between 1990 and 2021, males experienced greater increases in both incidence and prevalence compared with females. Male incidence increased by 31.9% (95% UI: −25.2 to 125.7), while female incidence increased by 14.3% (95% UI: −34.5 to 102.3). Male prevalence increased by 32.3% (95% UI: 5.7 to 65.0), whereas female prevalence increased by 12.8% (95% UI: −7.3 to 37.7). However, YLDs demonstrated divergent patterns, with males experiencing a 4.3% increase (95% UI: −41.8 to 86.2) and females showing a 3.4% decline (95% UI: −48.7 to 81.3). Sex-specific trends are illustrated in Figure 1 and detailed in Table 1.

### 3.3. SCI Burden by Cause: Transport-Related Versus Non-Transport Unintentional Injuries

Non-transport unintentional injuries accounted for the predominant cause of SCI burden throughout the study period, accounting for 75.5% of the total age-standardized prevalence and 74.8% of YLDs in 2021. The age-standardized prevalence of non-transport SCI increased from 175.6 per 100,000 (95% UI: 129.1 to 236.9) in 1990 to 219.3 per 100,000 (95% UI: 159.1 to 296.4) in 2021, representing a 24.9% increase (95% UI: 1.1 to 54.4). In contrast, transport-related SCI prevalence increased from 58.2 per 100,000 (95% UI: 41.2 to 80.4) in 1990 to 71.2 per 100,000 (95% UI: 49.9 to 98.4) in 2021, representing a 22.3% increase (95% UI: −5.1 to 56.8).

**Figure 2:**
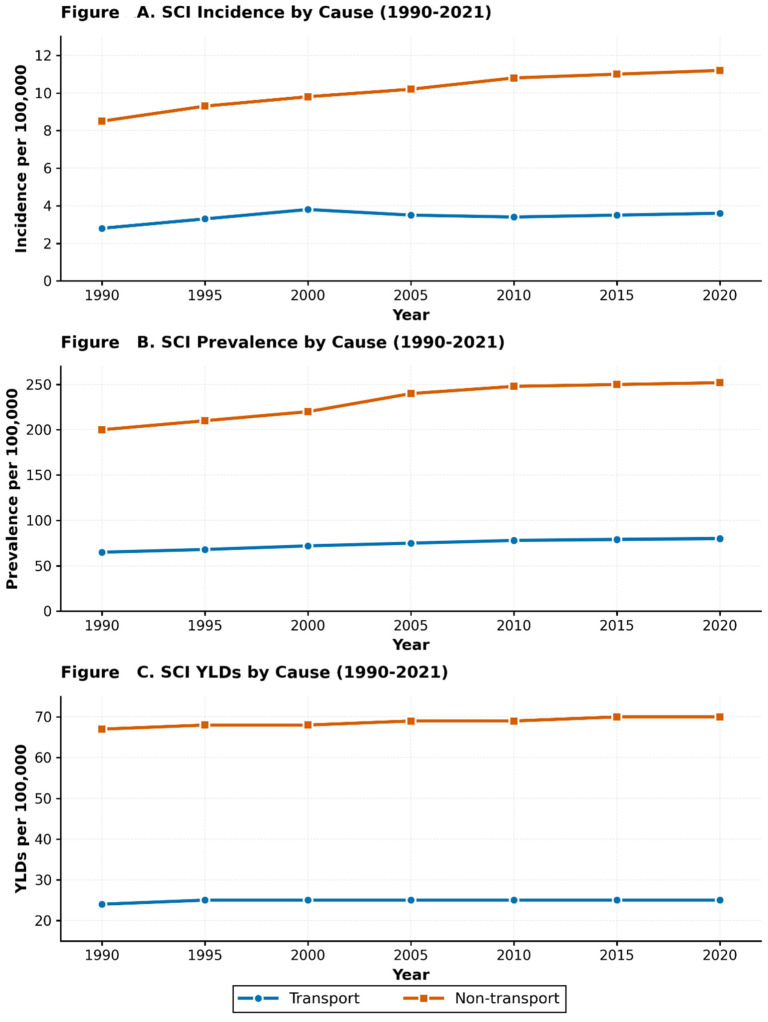
Cause-specific age-standardized spinal cord injury burden in Saudi Arabia, 1990–2021, comparing transport-related and non-transport unintentional injuries. (**A**) Incidence rates per 100,000 population. (**B**) Prevalence rates per 100,000 population. (**C**) Years lived with disability (YLD) rates per 100,000 population. Data are stratified by injury cause (transport-related versus non-transport unintentional injuries), with 95% confidence intervals.

YLDs attributable to non-transport injuries increased from 65.8 per 100,000 (95% UI: 45.0 to 93.2) in 1990 to 66.3 per 100,000 (95% UI: 44.9 to 94.9) in 2021, reflecting a minimal change of 0.8% (95% UI: −45.5 to 83.0). Transport-related YLDs increased from 21.7 per 100,000 (95% UI: 14.5 to 31.2) in 1990 to 22.4 per 100,000 (95% UI: 14.8 to 32.6) in 2021, representing a 3.2% increase (95% UI: −45.7 to 89.4). Temporal patterns by injury cause are presented in Figure 2 and Table 2.

### 3.4. Age- and Sex-Specific SCI Burden in 2021

In 2021, SCI burden exhibited substantial variation across age groups and between sexes (Table 3). SCI burden showed substantial variation across age groups and between sexes, characterized by a bimodal age distribution (young and older adults). SCI burden showed substantial variation across age groups and between sexes, characterized by a bimodal age distribution (young and older adults). The peak age-specific prevalence rate for any subgroup was observed among males aged ≥ 55 years, who experienced 668.4 per 100,000 (95% UI: 565.6 to 772.3) due to non-transport injuries. Females aged ≥ 55 years showed a non-transport prevalence of 274.0 per 100,000 (95% UI: 231.8 to 321.2). Prevalence rates remained elevated in the 15–49 age group, with males at 618.0 per 100,000 (95% UI: 451.5 to 831.4) and females at 211.4 per 100,000 (95% UI: 152.5 to 284.9). Among older adults aged 70 years and above, prevalence rates were substantially lower, with males at 67.4 per 100,000 (95% UI: 47.5 to 92.8) and females at 41.7 per 100,000 (95% UI: 29.0 to 58.2).

YLDs were highest among working-age males (15–49 years). The peak age-specific YLD rate (176.5 per 100,000, 95% UI: 124.5 to 233.2) was observed among males aged ≥55 years, specifically attributable to non-transport unintentional injuries.

**Figure 3:**
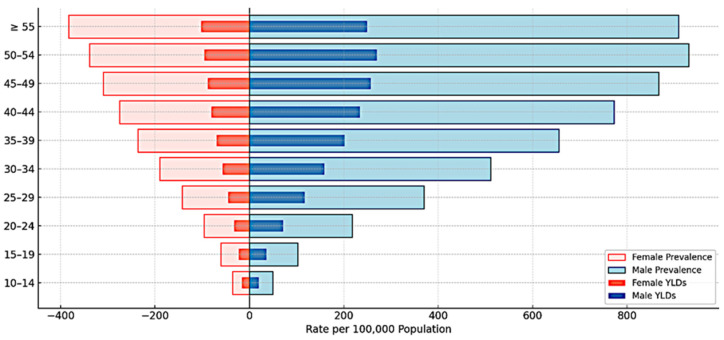
Age- and Sex-Specific Prevalence and Years Lived with Disability (YLD) Rates for Spinal Cord Injury in Saudi Arabia, 2021. Data are presented with 95% uncertainty intervals for males and females across five-year age cohorts.

**Figure 4:**
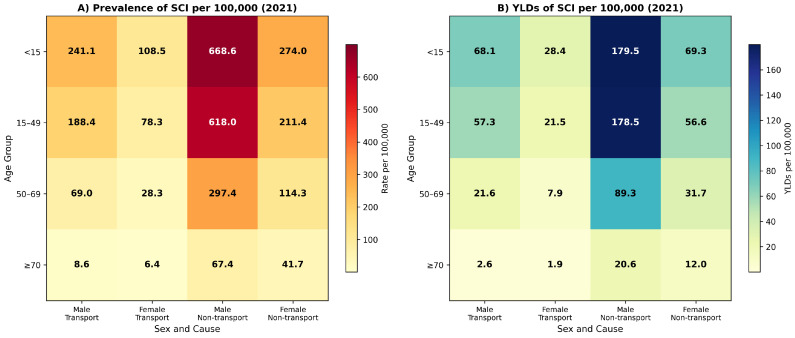
Age- and sex-specific spinal cord injury burden by cause in Saudi Arabia, 2021. (**A**) Prevalence rates per 100,000 population for transport-related and non-transport unintentional injuries, stratified by age group and sex. (**B**) Years lived with disability (YLD) rates per 100,000 population for transport-related and non-transport unintentional injuries, stratified by age group and sex. Heat maps display the distribution of burden across demographic groups and injury causes.

Non-transport unintentional injuries consistently contributed a larger proportion of SCI burden than transport-related injuries across all age groups and both sexes in 2021. Among males aged 15–49 years, non-transport injuries accounted for 76.7% of prevalence and 75.7% of YLDs, while transport-related injuries accounted for the remaining 23.3% and 24.3%, respectively. Among females in the same age group, non-transport injuries accounted for 73.0% of prevalence and 72.5% of YLDs. This pattern persisted across all age groups, with non-transport injuries consistently accounting for approximately three-quarters of the total SCI burden. Age- and sex-specific distributions are illustrated in Figure 3 and Figure 4.

## 4. Discussion

This study provides the first comprehensive, population-based analysis of three-decade trends in SCI burden across Saudi Arabia using standardized GBD 2021 methodology. Our findings reveal three principal patterns: first, age-standardized SCI incidence and prevalence demonstrated increases of approximately 25% between 1990 and 2021, although wide uncertainty intervals limit definitive conclusions about trend direction; second, non-transport unintentional injuries, predominantly falls, have emerged as the leading cause of SCI burden, accounting for approximately 75% of disability burden in 2021; and third, substantial disparities persist across sex and age groups, with males bearing twice the burden of females and young adults experiencing the highest incidence rates, while the peak cumulative prevalence burden is concentrated in the older adults (≥55 years) demographic, driven by improved survival and age-related fall risk.

The observed increase in age-standardized SCI incidence from 10.7 to 13.4 per 100,000 population represents a 25.0% rise over 31 years. However, the uncertainty interval crosses zero, indicating that the trend’s direction cannot be determined with statistical certainty. Similarly, prevalence increased from 233.8 to 290.5 per 100,000, representing a 24.3% increase with a narrower uncertainty interval that does not cross zero, suggesting greater confidence in this upward trajectory. These estimates align with recent global analyses reporting stable or slightly increasing SCI trends in middle-income countries [24]. The divergence between increasing prevalence and relatively stable YLDs suggests improvements in survival without corresponding reductions in disability severity, potentially reflecting enhanced acute care and trauma management under Vision 2030 health reforms [14,18].

The male-to-female ratio of approximately 2:1 observed in our study is consistent with global patterns documented across high- and middle-income countries, and in the southern region of Saudi Arabia [3,24,26]. This sex disparity likely reflects multiple factors, including higher occupational injury exposure among males, greater engagement in high-risk activities such as motor vehicle operation and construction work, and sociocultural factors influencing mobility patterns and risk-taking behaviors [8,26]. The historical prohibition on women driving in Saudi Arabia until 2017 likely contributed to lower transport-related SCI rates among females [15]. Future surveillance should monitor whether female injury patterns shift following policy changes that expand women’s participation in driving and workforce activities.

The predominance of non-transport unintentional injuries, particularly falls, represents a critical epidemiological transition. Non-transport injuries accounted for 75.5% of SCI prevalence and 74.8% of YLDs in 2021, substantially exceeding transport-related causes. This pattern contrasts with earlier hospital-based studies from 2003 to 2018 that identified motor vehicle collisions as the leading cause, accounting for 80–85% of traumatic SCI cases [11,12,13,17]. Several factors may explain this apparent shift. First, Vision 2030 traffic safety initiatives, including automated enforcement systems and enhanced trauma care networks, appear to have contributed to a 35% reduction in road traffic fatalities between 2016 and 2021, according to preliminary WHO data [14,17]. Second, rapid construction expansion and infrastructure development under Vision 2030 economic diversification programs have increased occupational fall exposure among workers [16,27]. Third, demographic aging has expanded the population vulnerable to low-energy falls, with life expectancy increasing from 70.6 to 76.4 years between 2000 and 2021 [10,28]. This demographic transition has been accompanied by documented increases in fall-related injury burden, with GBD data showing that fall incidence among Saudi older adults aged 55 years and above increased 69% for males and 30% for females between 1990 and 2019, reaching prevalence rates exceeding 45,000 per 100,000 population among older men [29]. Fourth, methodological differences between facility-based case series and population-level GBD estimates may contribute to apparent discrepancies, as hospital studies capture acute traumatic presentations. At the same time, GBD modeling incorporates broader community-based patterns, including chronic and progressive non-traumatic cases [30].

The age-specific burden distribution reveals concerning patterns requiring targeted interventions. The highest prevalence rates occurred among individuals aged younger than 15 years and those aged 15–49 years, suggesting that SCI predominantly affects individuals during their most productive life stages. Young adult males aged 15–49 years experienced prevalence rates of 618.0 per 100,000 and YLD rates of 178.5 per 100,000, reflecting substantial long-term disability burden and economic productivity loss. Among older adults aged 70 years and above, lower SCI prevalence rates likely reflect competing mortality risks despite documented increases in underlying fall incidence; recent GBD analysis demonstrated that fall-related injury burden among Saudi older adults increased substantially through 2019, with males experiencing prevalence rates exceeding 45,000 per 100,000 population [29], consistent with established SCI epidemiology demonstrating higher case fatality in older populations [30,31]. The elevated burden among children and adolescents aged younger than 15 years, with male prevalence reaching 668.4 per 100,000, underscores the need for pediatric injury prevention programs and age-appropriate rehabilitation services [32,33].

Our findings have important implications for Saudi Arabia’s Vision 2030 health transformation agenda and national injury prevention strategies. First, the shift toward non-transport injuries necessitates diversified prevention approaches beyond traditional road safety measures. Construction site safety regulations, occupational health standards, and fall prevention programs targeting both workers and older adults require strengthening [27,34]. Second, the persistent male predominance and concentration of burden among working-age populations highlight workplace opportunities-based interventions and targeted safety campaigns [26,33]. Third, the relatively stable YLD burden despite increasing prevalence suggests that current rehabilitation services may be reaching affected individuals, though gaps in vocational rehabilitation and community reintegration persist [33,35,36]. Fourth, the absence of a national SCI registry remains a critical limitation for comprehensive surveillance, quality improvement initiatives, and resource allocation [18,32].

International comparisons provide valuable context for Saudi Arabia’s SCI burden. Our estimated age-standardized prevalence of 290.5 per 100,000 in 2021 exceeds recent estimates from neighboring Middle Eastern countries. Still, it remains lower than rates reported in some high-income nations with mature trauma systems and extended survival patterns [20,24]. The predominance of non-transport injuries in Saudi Arabia parallels trends observed in aging populations globally, where falls increasingly contribute to SCI burden [30]. However, the persistent contribution of transport-related injuries, accounting for approximately 25% of the burden, remains higher than proportions observed in countries with well-established traffic safety infrastructure [3,31].

Several methodological considerations warrant acknowledgment. The GBD 2021 estimates for Saudi Arabia are derived from synthesized data sources using Bayesian modeling rather than direct national surveillance, introducing inherent uncertainty reflected in wide confidence intervals. Where Saudi-specific primary data were limited, estimates incorporated regional patterns adjusted for country-specific covariates. The wide uncertainty intervals, particularly for incidence trends, restrict our ability to make definitive conclusions about temporal changes. Hospital-based studies from Saudi trauma centers have reported higher proportions of transport-related injuries than our GBD-based estimates, likely reflecting selection bias toward acute traumatic presentations in facility-based case series. The GBD methodology captures broader population patterns, including community-based cases and progressive non-traumatic etiologies that may not present to specialized trauma centers. Saudi Arabia has demonstrated capacity for comprehensive disability surveillance through its 2016 national demographic survey, which successfully captured disability prevalence, type, and severity data across all regions using standardized methodology [37]. This existing epidemiological infrastructure provides a proven foundation for developing condition-specific surveillance systems. Additionally, improvements in case ascertainment, diagnostic capabilities, and reporting completeness over the 31-year study period may contribute to apparent increases independent of actual epidemiological changes. These limitations underscore the critical need to establish a comprehensive national SCI registry with standardized case definitions and systematic data collection protocols to enable more precise trend monitoring and policy evaluation.

Future research priorities should focus on several key areas. Establishing a population-based SCI registry would enable accurate incidence calculation, detailed etiology assessment, and longitudinal outcomes tracking [18,38,39]. Prospective cohort studies examining functional recovery trajectories, healthcare utilization patterns, and economic impacts would inform resource allocation and service planning [33,36]. Investigating the effects of Vision 2030 policy, including traffic safety interventions and expanded rehabilitation services, requires rigorous evaluation using interrupted time-series or quasi-experimental designs. Qualitative research exploring the lived experiences of individuals with SCI and their families would identify barriers to accessing care, participating in the community, and maintaining quality of life [39]. Finally, comparative effectiveness studies of rehabilitation interventions adapted to Saudi Arabian healthcare contexts would optimize clinical practice and resource utilization [34,36].

## 5. Conclusions

This population-based analysis reveals that Saudi Arabia experienced substantial increases in SCI burden between 1990 and 2021, with age-standardized prevalence rising by 24.3% to 290.5 per 100,000 population. The most striking finding is the fundamental etiological transition from transport-dominated injury patterns to non-transport unintentional injuries, predominantly falls, which now account for three-quarters of disability burden. This shift contrasts sharply with earlier hospital-based studies, which reported motor vehicle collisions as the cause in 80–85% of cases, suggesting that Vision 2030 road safety initiatives may be reducing transport injuries while construction expansion and demographic aging have elevated fall-related risks. The persistent twofold male predominance and concentration of burden among working-age populations and older adults underscore the substantial economic and social consequences of this condition.

These findings mandate a strategic reorientation of injury prevention policies aligned with Saudi Arabia’s Vision 2030 health transformation agenda. Comprehensive prevention programs must extend beyond road safety to encompass construction site regulations, occupational health standards, and fall prevention initiatives targeting both workers and aging populations. Establishing a national SCI registry is the highest priority, as the absence of a standardized surveillance infrastructure impedes precise burden estimation, policy evaluation, and evidence-based resource allocation. Enhanced rehabilitation services with vocational reintegration programs, particularly for young adults during peak productivity years, require urgent expansion to address long-term disability consequences. Success demands coordinated, multi-sectoral action across health, transportation, labor, and urban planning to mitigate the growing burden and improve outcomes for affected individuals and families in this rapidly developing nation.

## Figures and Tables

**Table 1:** Age-standardized incidence, prevalence, and years lived with disability (YLD) rates for spinal cord injury in Saudi Arabia, 1990–2021, stratified by sex.

Measure	Sex	1990 Rate (95% UI)	2005 Rate (95% UI)	2021 Rate (95% UI)	% Change 1990–2021 (95% UI) *
Incidence	Male	15.2 (11.9, 19.5)	17.7 (13.6, 23.4)	20.1 (15.2, 26.7)	31.9 (–21.8, 124.0)
	Female	7.5 (5.4, 10.5)	8.1 (5.7, 11.5)	7.9 (5.4, 11.3)	4.1 (–48.7, 110.0)
	Both	12.1 (9.4, 15.7)	13.7 (10.3, 18.2)	15.1 (11.3, 20.3)	25.0 (–28.3, 116.8)
Prevalence	Male	340.3 (308.0, 378.2)	376.5 (337.5, 422.8)	450.1 (404.2, 507.7)	32.3 (6.9, 64.9)
	Female	181.6 (162.5, 205.2)	184.4 (164.0, 209.2)	185.9 (165.4, 213.0)	2.3 (–19.4, 31.0)
	Both	278.2 (253.4, 310.0)	298.7 (270.4, 333.2)	345.9 (312.4, 388.7)	24.3 (0.8, 53.4)
YLD	Male	119.2 (87.6, 152.3)	120.4 (85.6, 156.7)	128.6 (89.3, 168.8)	7.9 (–41.3, 92.6)
	Female	60.2 (42.8, 79.5)	55.6 (39.5, 74.5)	49.6 (34.1, 65.3)	–17.6% (–57.1, 52.6)
	Both	96.1 (69.7, 123.2)	94.2 (67.4, 122.2)	97.5 (68.4, 128.1)	1.4% (–44.5, 83.9)

The data are presented as means with 95% uncertainty intervals, with rates and percentages rounded to one decimal place. Abbreviations: UI = uncertainty interval; YLD = years lived with disability. * Percent-change UIs are approximated using the ratio of 95% UI bounds for 1990 and 2021.

**Table 2:** Cause-specific age-standardized spinal cord injury incidence, prevalence, and years lived with disability (YLD) rates in Saudi Arabia, 1990 versus 2021: comparison of transport-related and non-transport unintentional injuries.

Measure	Causes	1990 Rate (95% UI)	2021 Rate (95% UI)	Rate Ratio 1990 (T:NT)	Rate Ratio 2021 (T:NT)	% Change 1990–2021 (95% UI)	AAPC 1990–2021 (95% UI)
Incidence	Transport	3.2 (2.3, 4.6)	3.7 (2.6, 5.2)	0.38	0.34	14.6 (–43.7, 132.1)	0.71 (0.33, 1.09)
	Non-transport	8.5 (5.9, 12.3)	10.9 (7.4, 16.3)	2.61	2.94	28.8 (–39.8, 177.3)	0.87 (0.74, 1.01)
Prevalence	Transport	68.5 (61.9, 77.3)	76.9 (68.4, 88.9)	0.35	0.31	12.3 (–11.6, 43.6)	0.59 (0.31, 0.87)
	Non-transport	197.6 (175.3, 226.3)	251.5 (220.8, 291.1)	2.89	3.27	27.3 (–2.4, 66.1)	0.79 (0.59, 0.98)
YLD	Transport	24.0 (17.1, 31.2)	22.3 (15.5, 29.8)	0.35	0.32	–7.2 (–50.3, 74.0)	–0.06 (–0.30, 0.19)
	Non-transport	67.8 (48.5, 87.2)	70.2 (48.7, 93.2)	2.82	3.15	3.5 (–44.2, 92.3)	0.09 (–0.08, 0.26)

The data are presented as means with 95% uncertainty intervals, with rates, percentages, and ratios reported to 1 and 2 decimal places, respectively. Abbreviations: UI = uncertainty interval; YLD = years lived with disability; T = transport-related; NT = non-transport unintentional; AAPC = average annual percent change.

**Table 3:** Age-, sex-, and cause-specific prevalence and years lived with disability (YLD) rates for spinal cord injury in Saudi Arabia, 2021: comparison of transport-related and non-transport unintentional injuries.

Measure	Age Group	Male Transport Rate (95% UI)	Female Transport Rate (95% UI)	Male Non-Transport Rate (95% UI)	Female Non-Transport Rate (95% UI)	Rate Ratio Transport	Rate Ratio Non-Transport	Δ Rate Ratio (T-NT)
Prevalence	10–14	5.1 (4.1, 6.1)	4.7 (3.9, 5.7)	44.7 (34.8, 57.0)	31.2 (23.8, 42.7)	1.07	1.43	–0.36
	15–19	12.1 (10.3, 14.5)	8.2 (6.9, 9.7)	90.1 (71.5, 116.2)	52.2 (40.5, 70.7)	1.48	1.72	–0.25
	20–24	35.3 (28.0, 43.6)	16.1 (13.2, 20.4)	182.8 (144.8, 235.3)	80.1 (62.7, 106.9)	2.19	2.28	–0.09
	25–29	67.7 (54.2, 85.4)	27.6 (22.0, 35.3)	302.4 (229.7, 393.8)	114.1 (89.2, 152.4)	2.45	2.65	–0.20
	30–34	104.1 (84.4, 127.6)	41.2 (32.3, 52.6)	407.0 (329.7, 514.4)	148.7 (119.8, 196.8)	2.53	2.74	–0.21
	35–39	139.9 (113.6, 170.0)	56.0 (44.3, 70.9)	515.3 (424.9, 633.9)	179.8 (145.5, 230.7)	2.50	2.87	–0.37
	40–44	173.5 (144.2, 209.9)	71.3 (58.3, 89.3)	599.4 (504.9, 719.7)	203.8 (166.1, 255.5)	2.43	2.94	–0.51
	45–49	206.2 (174.9, 241.1)	86.1 (69.9, 104.6)	661.0 (566.0, 784.2)	223.0 (182.9, 274.7)	2.39	2.96	–0.57
	50–54	234.1 (203.8, 272.3)	99.8 (81.7, 119.3)	696.5 (591.6, 807.7)	238.9 (197.4, 288.9)	2.35	2.91	–0.57
	≥ 55	241.1 (212.7, 275.5)	108.5 (92.5, 127.8)	668.4 (565.6, 772.3)	274.0 (231.8, 321.2)	2.22	2.44	–0.22
YLD	10–14	1.5 (0.6, 2.6)	1.4 (0.5, 2.6)	13.5 (5.5, 23.8)	9.0 (3.3, 17.4)	1.14	1.50	–0.36
	15–19	3.8 (2.1, 6.2)	2.4 (1.0, 4.0)	27.7 (14.3, 45.8)	15.0 (6.0, 27.7)	1.63	1.84	–0.21
	20–24	11.1 (6.7, 17.0)	4.6 (2.3, 7.7)	55.4 (31.8, 87.7)	22.5 (11.1, 39.1)	2.42	2.46	–0.04
	25–29	21.2 (13.6, 30.9)	7.8 (4.2, 12.7)	91.0 (55.0, 142.9)	31.9 (17.2, 51.3)	2.70	2.85	–0.15
	30–34	32.4 (20.2, 48.1)	11.4 (6.7, 17.6)	121.4 (75.4, 181.2)	40.7 (22.7, 62.9)	2.84	2.99	–0.15
	35–39	43.6 (28.6, 61.1)	15.5 (9.2, 24.1)	153.3 (97.2, 221.0)	48.9 (28.7, 75.8)	2.81	3.14	–0.33
	40–44	53.3 (35.4, 75.8)	19.8 (11.7, 28.8)	175.4 (116.0, 248.7)	55.1 (33.7, 81.5)	2.69	3.18	–0.50
	45–49	62.6 (41.2, 87.0)	23.6 (14.7, 34.6)	189.9 (127.3, 266.9)	59.4 (37.5, 88.3)	2.65	3.20	–0.55
	50–54	69.7 (46.2, 95.7)	27.2 (16.7, 38.9)	195.3 (131.5, 270.4)	63.0 (38.3, 91.9)	2.56	3.10	–0.54
	≥ 55	68.1 (48.3, 91.6)	28.4 (19.3, 39.0)	176.5 (124.5, 233.2)	69.3 (46.7, 93.7)	2.39	2.55	–0.15

The data are presented as means with 95% uncertainty intervals, with rates, percentages, and ratios reported to 1 decimal place. Abbreviations: T = transport-related; NT = non-transport unintentional; UI = uncertainty interval; YLD = years lived with disability.

## Data Availability

All data used in this study are openly available from the Global Burden of Disease 2021 Results Tool at https://vizhub.healthdata.org/gbd-results/ (accessed on 20 October 2025).

## Supplementary Materials

The following supporting information can be downloaded at https://www.mdpi.com/article/10.3390/jcm14248836/s1. Table S1: GATHER checklist.

## Abbreviations

The following abbreviations are used in this manuscript:

AAPC	Average Annual Percent Change
DisMod-MR	Disease Modeling Meta-Regression
GATHER	Guidelines for Accurate and Transparent Health Estimates Reporting
GBD	Global Burden of Diseases
ICD	International Classification of Diseases
SCI	Spinal Cord Injury
STROBE	Strengthening the Reporting of Observational Studies in Epidemiology
TSCI	Traumatic Spinal Cord Injury
UIs	95% Uncertainty Intervals
WHO	World Health Organization
YLDs	Years Lived with Disability
