# Supplementary material for: Three Decades of Spinal Cord Injury in Saudi Arabia: Trends in Incidence, Prevalence, and Disability Outcomes
Source: J Clin Med. 2025 Dec 13;14(24):8836. doi: 10.3390/jcm14248836 (PMC12734008; doi:10.3390/jcm14248836)
Supplement: Supplementary file 1 [file jcm-14-08836-s001.zip › jcm-4018032-supplementary.pdf]

Supplementary Table S1: GATHER Checklist (Guidelines for Accurate and Transparent Health Estimates Reporting)

GATHER Item	Requirement	Fulfillment in Manuscript	Compliance
**1. Define indicator(s), population(s) and time period(s) of estimates**	Indicators, population, and time period should be specified.	The study estimates age-standardized incidence, prevalence, and Years Lived with Disability (YLDs) for Spinal Cord Injury (SCI) in the Saudi Arabia population from 1990 to 2021.	Fully Compliant
**2. List the funding sources for the work**	List all sources of funding for the study.	Funding was provided by the Deanship of Graduate Studies and Scientific Research at Qassim University, grant number 2025–QU–APC.	Fully Compliant
**3. Describe how the data were identified and accessed**	Detail the process for finding and obtaining the data.	Data were extracted from the publicly accessible GBD 2021 Results Tool (https://vizhub.healthdata.org/gbd-results/), accessed on 20 October 2025.	Fully Compliant
**4. Specify inclusion and exclusion criteria**	Define the criteria used to select the data for the analysis.	Inclusion: Traumatic SCI classified by ICD codes ICD-N-33 and ICD-N-34 (below the cervical level) and stratified by external cause (transport-related V00–V99 and non-transport unintentional W00–W19, X00–X59). Exclusion: Intentional causes (violence, self-harm) and non-traumatic etiologies.	Fully Compliant
**5. Provide information on all included data sources and their main characteristics**	Describe the type and volume of data used in the model.	Estimates relied on the GBD 2021 synthesis of published literature, systematic reviews, vital registration systems, hospital records, survey data, Ministry of Health reports, and regional NAM&E pattern data.	Fully Compliant
**6. Identify and describe any input data that have potentially**	Discuss data quality issues.	The manuscript explicitly discusses that historical hospital-based SCI studies (reporting 80–85% transport etiology) reflect selection bias toward acute	Fully Compliant

**important biases.**		trauma centers and have temporal limitations (2003–2018).	
**7. Describe and give sources for any other data inputs**	Include auxiliary data or covariates.	Estimates were informed by regional North Africa and Middle East (NAME) pattern data adjusted for country-specific covariates, including healthcare access, injury risk factors, and demographic characteristics.	Fully Compliant
**8. Provide all data inputs in a format from which data can be efficiently extracted**	Make input data easily accessible.	The GBD estimates, which serve as the study's input data, are publicly available via the GBD 2021 Results Tool. The authors do not supply the raw data used by the GBD model itself.	Partially Compliant
**9. Provide a conceptual overview of the data analysis method**	Explain the overall analytical approach.	The conceptual approach is a population-based trend analysis using the Disease Modelling Meta-Regression (DisMod-MR) 2.1 Bayesian framework, as used in the GBD 2021 study.	Fully Compliant
**10. Provide a detailed description of all steps of the analysis**	Detail calculations for the reported estimates.	Description includes the extraction of GBD rates, calculation of absolute percentage change, Average Annual Percentage Change (AAPC), male-to-female rate ratios, and the use of non-overlapping 95% UIs for statistical difference.	Fully Compliant
**11. Describe how candidate models were evaluated and how the final model(s) were selected**	Describe the model selection process.	As a secondary analysis, the manuscript utilizes the established DisMod-MR 2.1 model but does not describe the specific evaluation or selection process conducted by the GBD 2021 collaborators.	Partially Compliant
**12. Provide the results of an evaluation of model performance and the results of any sensitivity analyses**	Report on model fit and robustness tests.	The manuscript reports the GBD model performance implicitly through the 95% UIs propagated by the Bayesian framework. No additional sensitivity analyses were performed by the authors (a limitation acknowledged in the discussion).	Partially Compliant
**13. Describe methods for calculating the uncertainty of the estimates**	Explain the source and calculation of uncertainty.	Uncertainty is quantified using 95% Uncertainty Intervals (UIs), reflecting the variation arising from data availability, modeling assumptions, and parameter estimation propagated	Fully Compliant

		through the DisMod-MR 2.1 Bayesian modeling approach.	
**14. State how the analytic or statistical code used to generate estimates can be accessed**	Provide instructions for obtaining code.	The R code used for data extraction, analysis, and visualization is available from the corresponding author upon reasonable request.	Fully Compliant
**15. Provide published estimates in a file format from which data can be efficiently extracted**	Make results easily usable by others.	The published estimates are presented in detailed tables (e.g., Tables 1, 2, and 3) and are also available in digital format via the GBD 2021 Results Tool.	Fully Compliant
**16. Report a quantitative measure of the uncertainty of the estimates**	Present numerical measures of uncertainty.	95% Uncertainty Intervals (UIs) are provided for all reported incidence, prevalence, YLD rates, and percentage changes.	Fully Compliant
**17. Interpret results in light of existing evidence. If updating a previous set of estimates, describe the reasons for changes in the estimates**	Compare findings with prior studies.	The discussion interprets findings against global trends, previous hospital-based Saudi studies (highlighting the shift away from transport predominance), and the context of Vision 2030 reforms.	Fully Compliant
**18. Discuss limitations of the estimates**	Clearly state data and methodological constraints.	Limitations are discussed, including reliance on GBD-modelled data, resulting in wide UIs; the inability to draw definitive conclusions about incidence trends; and discrepancies between GBD estimates and local facility data (selection bias).	Fully Compliant
